# Supplementary material for: Kondo screening in a Majorana metal
Source: Nat Commun. 2023 Nov 16;14:7405. doi: 10.1038/s41467-023-43185-3 (PMC10654600; doi:10.1038/s41467-023-43185-3)
Supplement: Supplementary file 1 — Supplementary Information [file 41467_2023_43185_MOESM1_ESM.pdf]

Supplementary Information for
Kondo screening in a Majorana metal

S. Lee[1,†], Y. S. Choi[2,†], S.-H. Do[3,†], W. Lee[1], C. H. Lee[4], M. Lee[5], M. Vojta[6], C. N. Wang[7], H. Luetkens[7], Z. Guguchia[7], and K.-Y. Choi[2,*]

[1] *Center for Artificial Low Dimensional Electronic Systems, Institute for Basic Science, Pohang 37673, Republic of Korea*

[2] *Department of Physics, Sungkyunkwan University, Suwon 16419, Republic of Korea*

[3] *Materials Science and Technology Division, Oak Ridge National Laboratory, Oak Ridge, Tennessee 37831, USA*

[4] *Department of Physics, Chung-Ang University, 84 Heukseok-ro, Seoul 06974, Republic of Korea*

[5] *National High Magnetic Field Laboratory, Los Alamos National Laboratory, Los Alamos, New Mexico 87545, USA*

[6] *Institut für Theoretische Physik, Technische Universität Dresden, 01062 Dresden, Germany*

[7] *Laboratory for Muon Spin Spectroscopy, Paul Scherrer Institute, Villigen PSI 5232, Switzerland*

[†] *These authors contributed equally: S. Lee, Y. S. Choi, S.-H. Do.*

[*] Corresponding author. Email: choisky99@skku.edu

Supplementary Note 1. EDX and XRD results of *α*-$Ru_{1-x}Cr_xCl_3$ single crystals

Supplementary Fig. 1 shows the distribution of Cr^{3+} concentration across several sites in the *α*-$Ru_{1-x}Cr_xCl_3$ (x=0.03) single crystal, represented by a histogram plot. The solid curve denotes a Gaussian fit to the distribution, yielding the Cr^{3+} concentration of x=0.030±0.0017. The inset shows a scanning electron microscopy image of the single crystal used for the measurements.

Supplementary Fig. 2a displays the XRD patterns of *α*-$Ru_{1-x}Cr_xCl_3$ single crystals at room temperature. Within the 2θ angle range of $10 \leq 2\theta \leq 80°$, we observe four diffraction peaks, indexed with (001), (003), and (004) in the $C2/m$ monoclinic setting. The (002) peak exhibits a very weak intensity. To determine the c-axis lattice parameter, we performed Rietveld refinement using FullProf software with the preferred orientation mode. The obtained lattice parameter c is plotted in Supplementary Fig. 2b. With increasing Cr^{3+} content, the c-axis lattice parameter shows a linear increment from 6.011 Å to 6.014 Å. These values are smaller than the previously reported results[1]. In addition, we plot the Rietveld refinement conducted by employing a two-phase model *α*-$RuCl_3$ + $CrCl_3$ in Supplementary Fig. 3. Apparently, *α*-$Ru_{1-x}Cr_xCl_3$ ($0 \leq x \leq 0.045$) exhibits no diffraction peaks for (002), (003), and (004) of $CrCl_3$. The estimated fraction of $CrCl_3$ is significantly less than 1 %. It is worth mentioning that our XRD patterns reveal no discernible peak splitting, which typically indicates phase segregation. The fit quality measures, including R-factors and χ^2, are indicated in the figure panels.

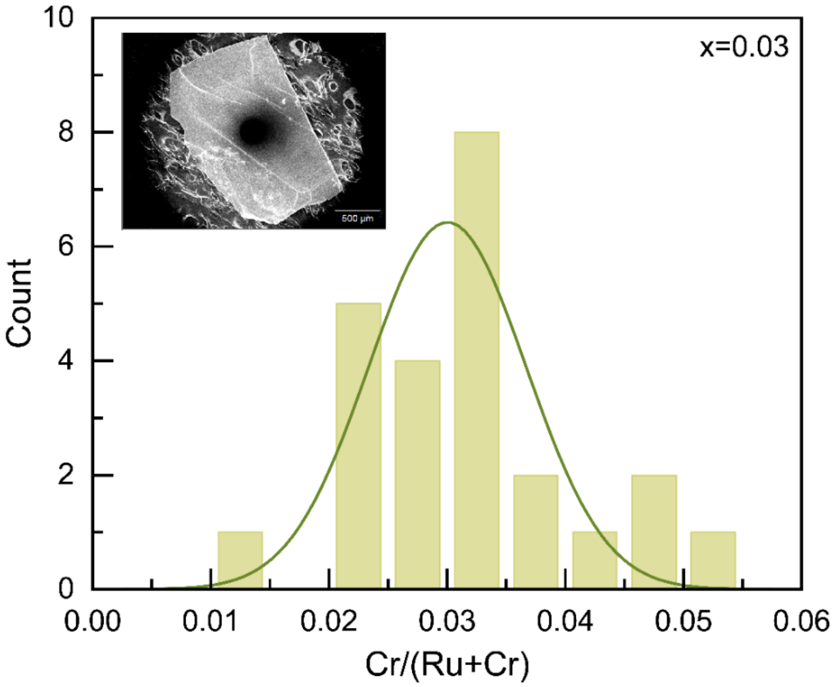

Supplementary Fig. 1 EDX results of *α*-$Ru_{1-x}Cr_xCl_3$ (x=0.03) single crystal.

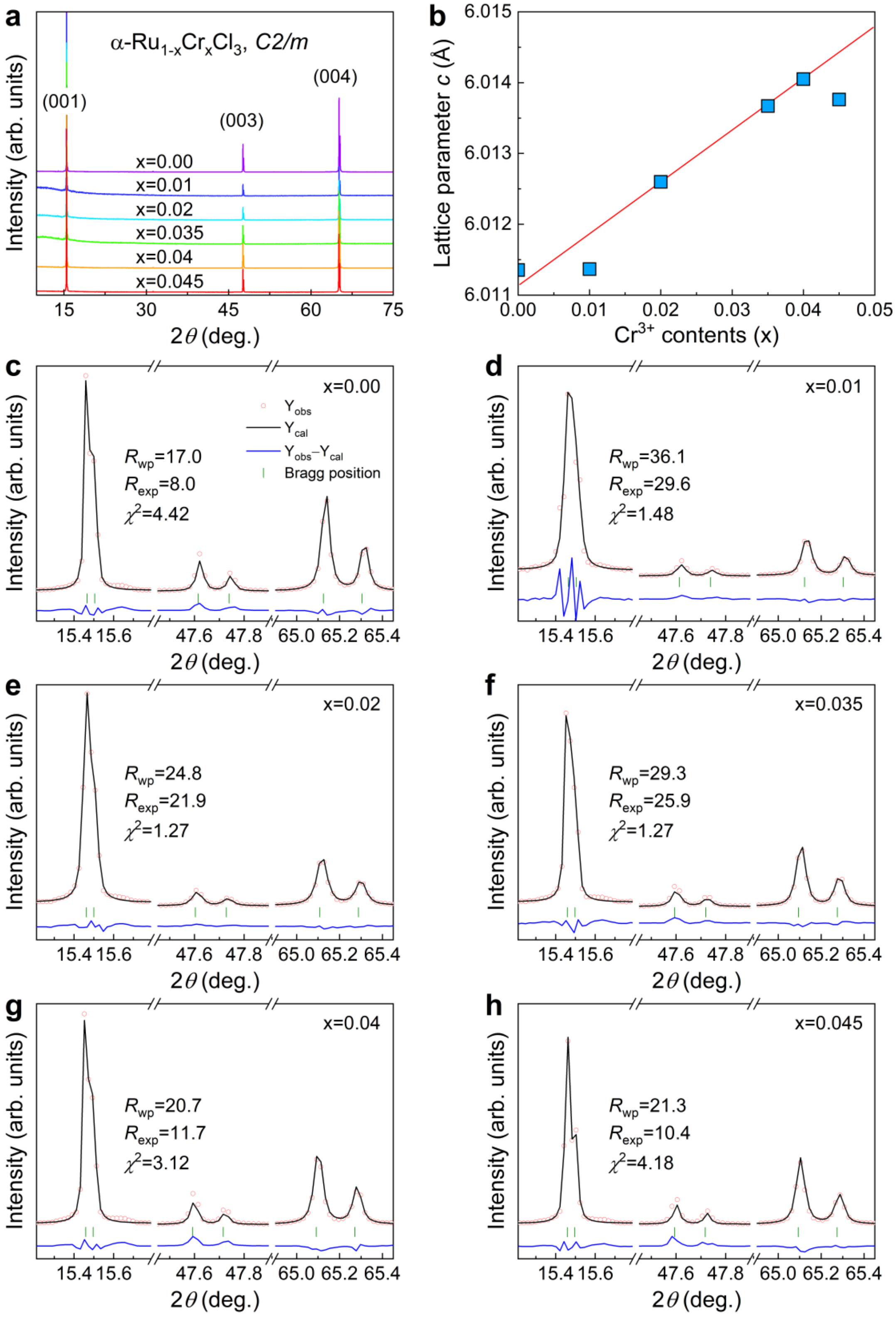

Supplementary Fig. 2 X-ray diffraction patterns and the *c*-axis lattice parameter. a X-ray diffraction patterns of α-$Ru_{1-x}Cr_xCl_3$ ($0 \leq x \leq 0.045$) at room temperature. The diffraction peaks are indexed with Miller (00*l*) indices. **b** *x* dependence of the *c*-axis lattice parameter obtained in the monoclinic (*C*2/*m*) setting. The red solid line is a guide to the eye. **c**-**h** Rietveld refinement of the X-ray diffraction of the *c*-axis oriented α-$Ru_{1-x}Cr_xCl_3$ single crystals.

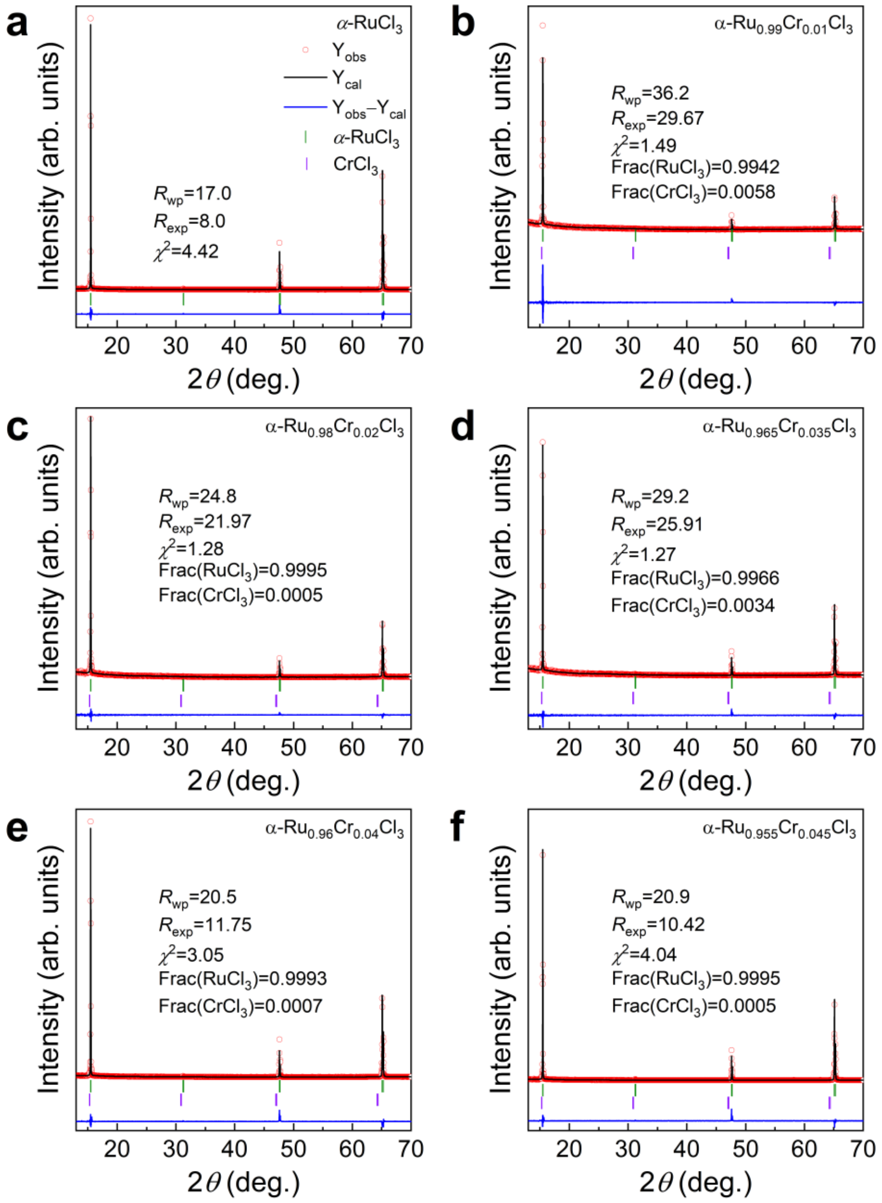

Supplementary Fig. 3 X-ray diffraction patterns with two-phase model *α*-$RuCl_3$ and $CrCl_3$. a-f X-ray diffraction patterns of α-$Ru_{1-x}Cr_xCl_3$ ($0 \leq x \leq 0.045$) with Rietveld refinements using two-phase model α-$RuCl_3$ + $CrCl_3$.

Supplementary Note 2. Composition dependence of the phonon parameters

In Supplementary Fig. 4, we present the room-temperature Raman spectra measured in (xu) polarization. In the (xu) scattering configuration, the incident light polarization is fixed to the a-axis, while the scattered light is unpolarized. According to the point group representation of $C2/m$ (poing group C_{2h}), the factor-group analysis predicts a total of 12 Raman-active modes $\Gamma = 6A_g + 6B_g$.

We observe the seven modes at 116 cm^{-1} [$A_g(1)+B_g(1)$], 161 cm^{-1} [$A_g(2)+B_g(2)$], 221.6 cm^{-1} [$A_g(3)+B_g(3)$], 271 cm^{-1} [$A_g(4)+B_g(4)$], 295.5 cm^{-1} [$A_g(5)+B_g(5)$], 311.5 cm^{-1} [$A_g(6)$], and 343 cm^{-1} [$B_g(6)$]. Overall, our Raman spectra are in good agreement with the previously reported data with respect to their spectral shape, energy, and symmetry[2,3]. We note that we can resolve additional weak phonon mode about 249 cm^{-1} (marked by the yellow bar). This mode is observed in pure $CrCl_3$[1], while showing no dependence on x. In these considerations, the 249 cm^{-1} peak can be interpreted as part of the Raman-active mode of $CrCl_3$ or can be attributed to symmetry reduction induced by stacking faults and strains. As the $A_g(1)+B_g(1)$ and $A_g(2)+B_g(2)$ modes mainly involve the displacement of the Ru atoms, they are coupled to a magnetic continuum, showing a Fano asymmetric profile. As evident from Supplementary Fig. 4b,c, the Fano modes are hardly affected by the Cr^{3+} substitution for Ru^{3+}. We further present the temperature dependence of the frequency, the FWHM, and normalized intensity of the $A_g(3)+B_g(3)$, $A_g(4)+B_g(4)$, $A_g(5)+B_g(5)$, and $A_g(6)$ phonons in Supplementary Fig. 5. The four modes exhibit no variation with the Cr^{3+} concentration. The observation confirms that the Cr^{3+}-for-Ru^{3+} substitution induces no discernible structural distortions.

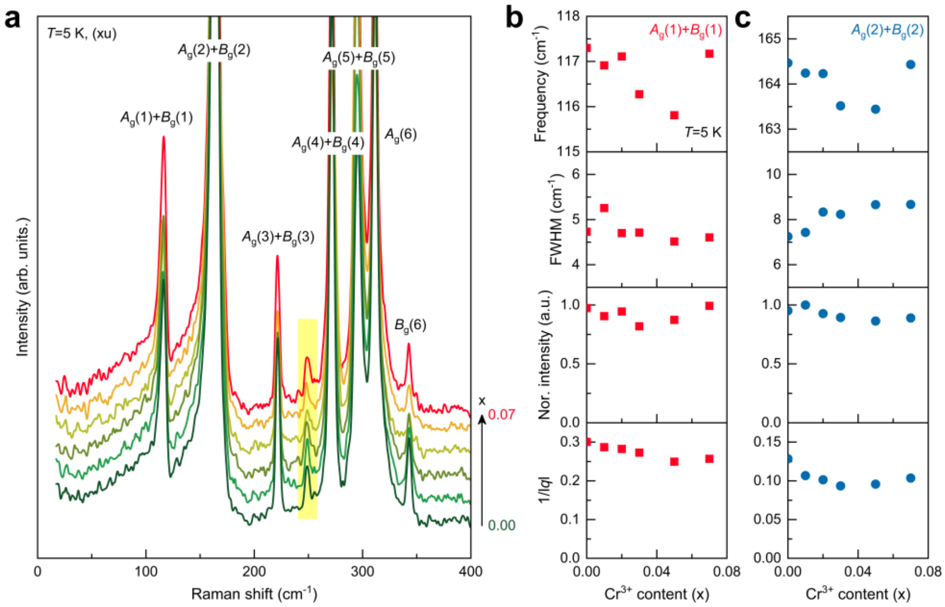

Supplementary Fig. 4 Raman spectra and the phonon parameters of the Fano modes. a Raman spectra of α-$Ru_{1-x}Cr_xCl_3$ measured at T=5 K in in-plane (xu) polarization. The characters on top of the peaks denote their symmetries. The yellow bar marks a symmetry-forbidden phonon at 249 cm^{-1}. **b**,**c** x dependence of the frequency, FWHM, normalized intensity, and the asymmetry parameter $1/|q|$ of the $A_g(1)+B_g(1)$ and $A_g(2)+B_g(2)$ modes.

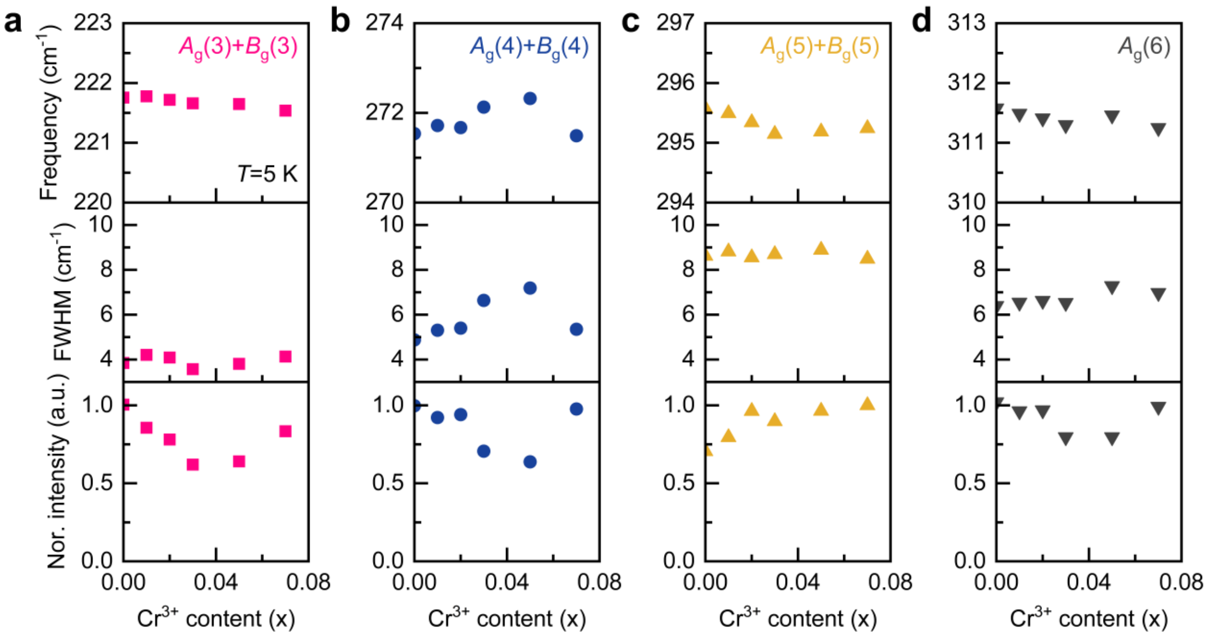

Supplementary Fig. 5 The phonon parameters as a function of Cr concentration. a-d Cr-content dependence of the frequency, FWHM, and normalized intensity of $A_g(3)+B_g(3)$, $A_g(4)+B_g(4)$, $A_g(5)+B_g(5)$, and $A_g(6)$ modes.

Supplementary Note 3. Magnetic and thermodynamic properties

Supplementary Fig. 6 shows the temperature dependence of the *dc* magnetic susceptibility $\chi(T)$ for $B//c$. The $\chi(T)$ data in the temperature range of $180<T<300$ K can be well-fitted by the Curie-Weiss law, as indicated by the dashed lines. Supplementary Fig. 7 summarizes the Néel temperature and magnetic parameters extracted from the dc magnetic susceptibility data. The AFM ordering temperature is slightly suppressed from T_N=6.5 K at x=0 to 5 K at x=0.03-0.07. The Curie-Weiss temperature Θ_{CW}^{ab} and the effective magnetic moment μ_{eff}^{ab} do not significantly vary with Cr^{3+} concentration. On the other hand, the large negative Θ_{CW}^{c} drastically decreases towards T=0 K and μ_{eff}^{c}=3 μ_B is reduced to 2.3 μ_B as x increases to 0.07.

Supplementary Fig. 8 exhibits the isothermal magnetization results for the field direction $B//ab$ and $B//c$ at T=2 K. With increasing x, $M_c(B)$ increases gradually, whereas $M_{ab}(B)$ varies hardly. Such disparate responses are in line with the dc magnetic susceptibility data (Fig. 2 in the main text). The first derivative of the magnetization dM/dB for $B//ab$ displays a weak peak at about 1-2 T and a broad maximum at B=6 T for x=0.00. The former is ascribed to the field-induced population of the magnetic domain in which the zigzag chains run along the field direction. On the other hand, the latter is linked to a spin-flop-like transition[4]. As the Cr content

increases, this broad maximum is gradually suppressed and nearly vanishes for $x \geq 0.03$. On the other hand, for $B//c$, the broad maximum appears and shifts to lower fields with increasing x. Note that, for x=0.04, the broad maximum in dM/dB is at around 0.6 T, comparable to the crossover field determined by high transverse field μSR (Supplementary Fig. 15). Therefore, we conclude that the Cr^{3+}- for-Ru^{3+} substitution modifies the magnetic domain and stacking patterns, thereby influencing the fluxes.

Supplementary Fig. 9 displays the subtracted magnetic susceptibility $\Delta\chi_c(T)=\chi_c(T)-\chi_c(T;x=0)$ in a semilogarithmic scale. We analyzed the $\Delta\chi_c(T)$ data by fitting them with the sum of logarithmic and power-law functions $\Delta\chi_c(T)\sim C_1\ln(D/T)+C_2T^{-\alpha}$. We found that $\Delta\chi_c(T)$ is dominated by the logarithmic (power-law) dependence in the low-temperature regime of $10<T<20$ K (high-temperature regime of $30<T<100$ K). The relative intensity C_1/C_2 between the logarithmic and power-law contributions is close to 1 and 0 for $10<T<20$ K and $30<T<100$ K, respectively. It is worth noting that the fits using $\Delta\chi_c(T)\sim C_1\ln(D/T)+C_2T^{-\alpha}$ for $10<T<100$ K do not provide proper description of the pristine sample, indicating that the logarithmic behavior emerges upon the introduction of magnetic impurities. Further, we observed that the exponent α slightly deviates from the Curie-Weiss-like behavior (α=1).

Supplementary Fig. 10 compares the subtracted magnetic susceptibility $\Delta\chi_c(T)$ with the theoretically predicted impurity susceptibility derived from the equivalent multichannel Kondo model. The multichannel Kondo model is characterized by the impurity spin S and the number of channels n. In the case of $n=2S$, the impurity spin is fully compensated by the conduction electron by forming a singlet state at low temperatures[5]. The impurity contribution to the magnetic susceptibility $\chi_{\mathrm{imp}}(T)$ is calculated by numerical simulations[5,6].

To obtain insights into the Kondo screening phenomenon in α-$Ru_{1-x}Cr_xCl_3$, we fit the $\Delta\chi_c(T)$ data using the theoretically predicted impurity susceptibility in the temperature range of $T_N<T<100$ K. As shown in Supplementary Fig. 10a-e, the behavior of $\Delta\chi_c(T)$ is qualitatively described with the equivalent multichannel Kondo effect in the temperature range of $T_N<T<T_K^{\mathrm{onset}}$. Nevertheless, deviations from the theoretical model become evident as the temperature is raised. This deviation may be attributed to additional contributions caused by the Cr-for-Ru substitution

From the fittings, we further extract the Kondo temperature T_K, as plotted in Supplementary Fig. 10f. It turns out that $T_K(x)$ is substantially smaller when compared to the onset temperature T_K^{onset} of the logarithmic dependence of $\Delta\chi_c(T)$. This discrepancy is ascribed to the irrelevance

of the equivalent multichannel Kondo model in describing the Kondo screening in α-$Ru_{1-x}Cr_xCl_3$. Instead, the Cr impurity in α-$Ru_{1-x}Cr_xCl_3$ is expected to be described by a S=3/2 three-channel Kondo model where two of the channels (p+ and p-) are equivalent, while the third (s) differs[7]. We note that the inequivalent three-channel Kondo model will still lead to full screening in the low-temperature limit, but the crossovers will be different from the model involving three equivalent screening channels. In the framework of inequivalent screening channels, the weaker screening channel determines the lower Kondo temperature (say T_K^{low}), below which $\Delta\chi_c(T)$ would reach saturation. In contrast, the stronger of the screening channels determines the higher Kondo temperature (say T_K^{high}), which is roughly the scale where the logarithmic behavior sets in. Then, we would have T_K^{low}~1 K and T_K^{high} ~10 K, and the remaining deviations may come from inappropriate fitting functions and the influence of vison dynamics.

Supplementary Figs. 11 and 12 provide additional information on the magnetic susceptibility and specific heat $C_m(T)$ of α-$Ru_{1-x}Cr_xCl_3$ (x=0.02, 0.04). For x=0.02, the low-T plateau in the T=15-50 K range observed in α-$RuCl_3$ (Fig. 3c in the main text) turns into a weak linear slope. Consequently, there are two linearly increasing regimes (Supplementary Fig. 11b). Combining the magnetic specific heat data for x=0.00 and 0.04 (Fig. 3), the emergence of the constant $C_m(T)/T$ term suggests the systematic modification of low-E magnetic density of states by the Cr substitution. On the other hand, this observation confirms that the addition of 2 % magnetic impurities expands the temperature window of metallic Majorana down to ~ 10 K.

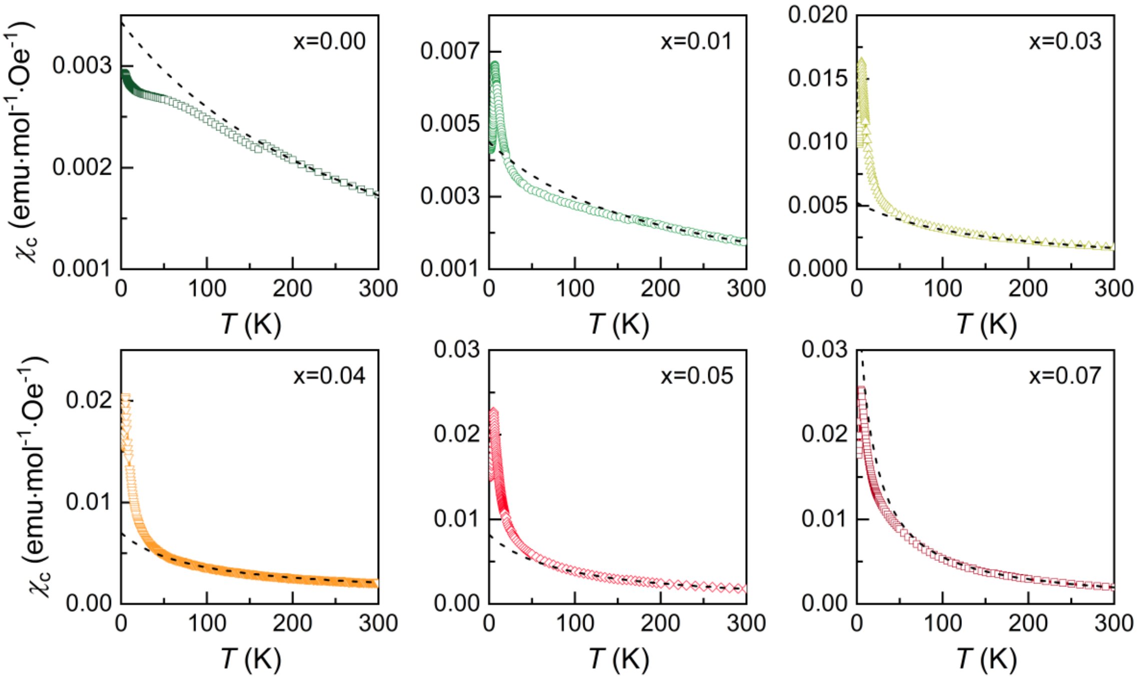

Supplementary Fig. 6 Temperature dependence of the magnetic susceptibility of *α*-$Ru_{1-x}Cr_xCl_3$ (x=0.00-0.07). Magnetic susceptibility as a function of temperature under the applied field of $B//c$=0.1 T in a semilog scale. The dashed lines denote the Curie-Weiss fittings to the data in the temperature range of T=180-300 K. The obtained parameters are summarized in Supplementary Fig. 7.

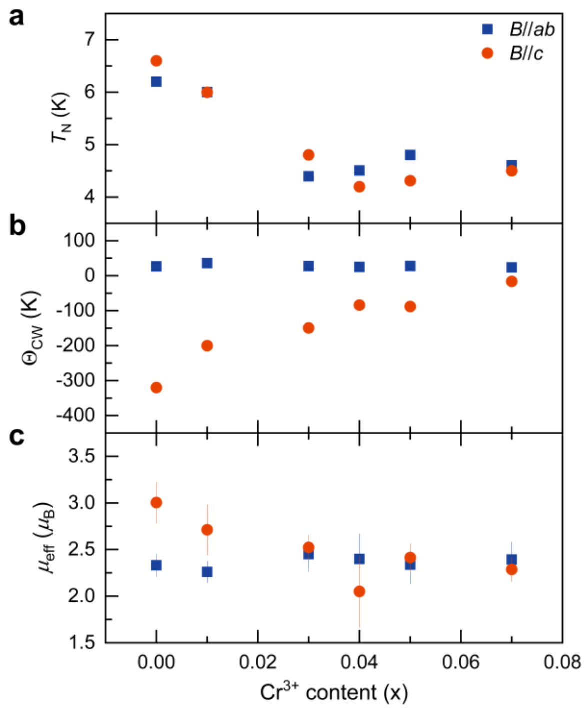

Supplementary Fig. 7 Néel temperature and magnetic parameters extracted from dc magnetic susceptibility data. a Néel temperature of *α*-$Ru_{1-x}Cr_xCl_3$ as a function of the Cr content (x). T_N is determined from the peak of $d(\chi T)/dT$. **b** x dependence of the Curie-Weiss temperature Θ_{CW} evaluated from the Curie-Weiss analysis of $\chi(T)$ data. With increasing x, Θ_{CW} is strongly repressed only for $B//c$ (orange circles), while Θ_{CW} for $B//ab$ (blue squares) hardly varies with x. **c**, Effective magnetic moment as a function of x. μ_{eff} for $B//c$ shows a systematic decrease with increasing x, while μ_{eff} for $B//ab$ remains nearly constant. Error bars represent standard deviation of the Curie-Weiss fit parameters.

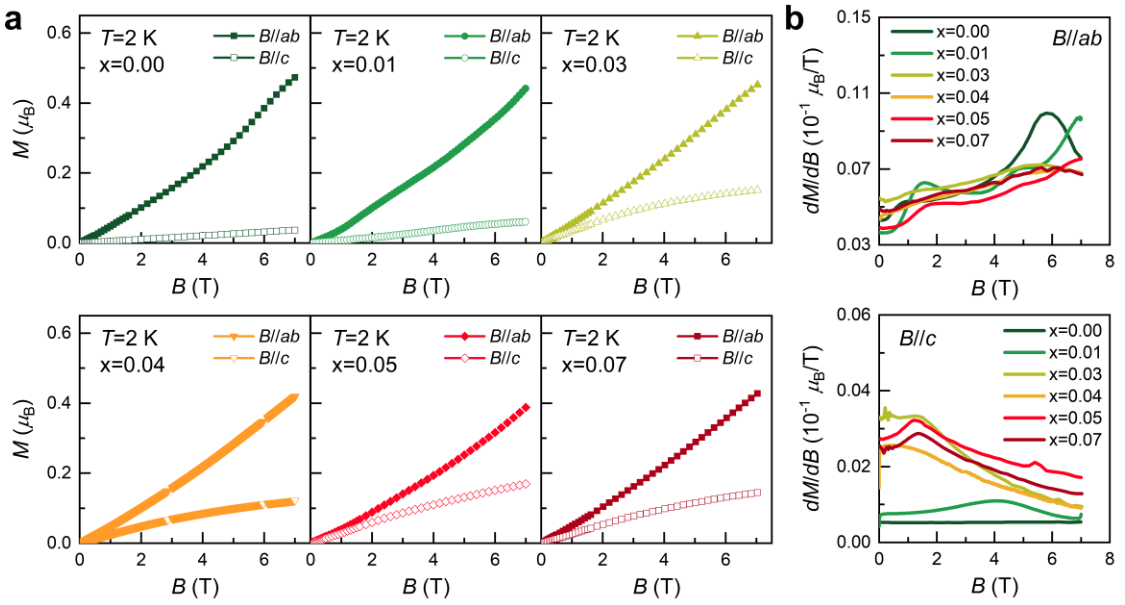

Supplementary Fig. 8 Isothermal magnetization data. a Isothermal magnetization at T=2 K of α-$Ru_{1-x}Cr_xCl_3$ (x=0–0.07) along the *ab*-plane (closed symbols) and *c*-axis (open symbols). **b** Field derivatives of the magnetization dM/dB for the field directions of $B//ab$ (upper panel) and $B//c$ (lower panel) at T=2 K.

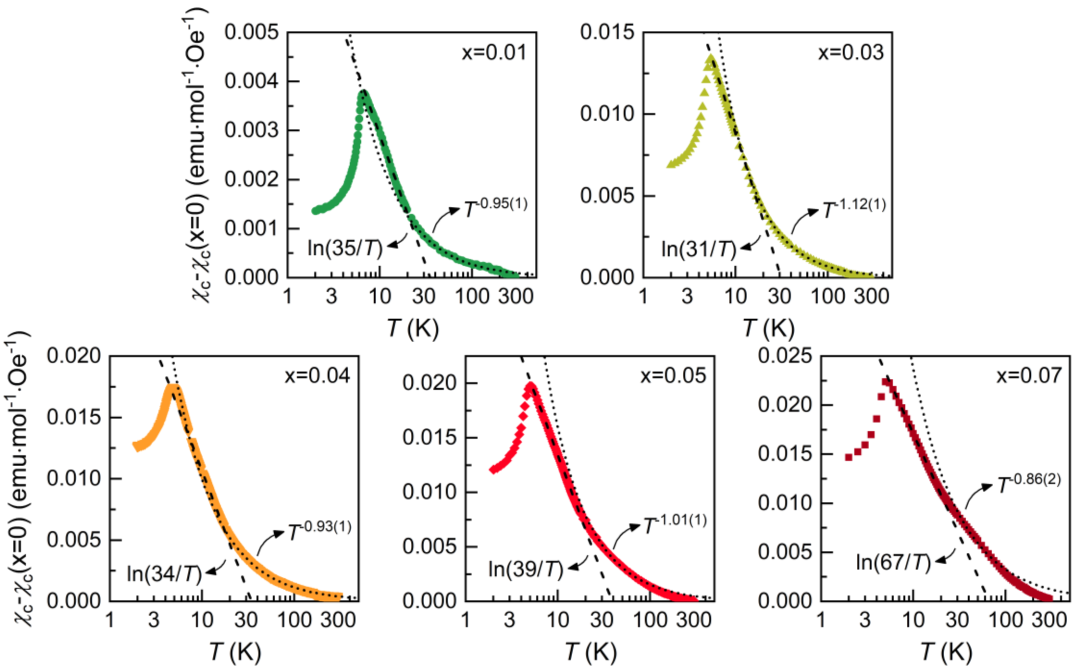

Supplementary Fig. 9 Logarithmic and power behavior of the magnetic susceptibility. Temperature dependence of the subtracted magnetic susceptibility for $B//c$=0.1 T in a semilog scale. The dashed and dotted lines represent the logarithmic ($\ln(D/T)$) and power-law (T^{-a}) behaviors at low (T=10-20 K) and high (T=30-100 K) temperatures, respectively.

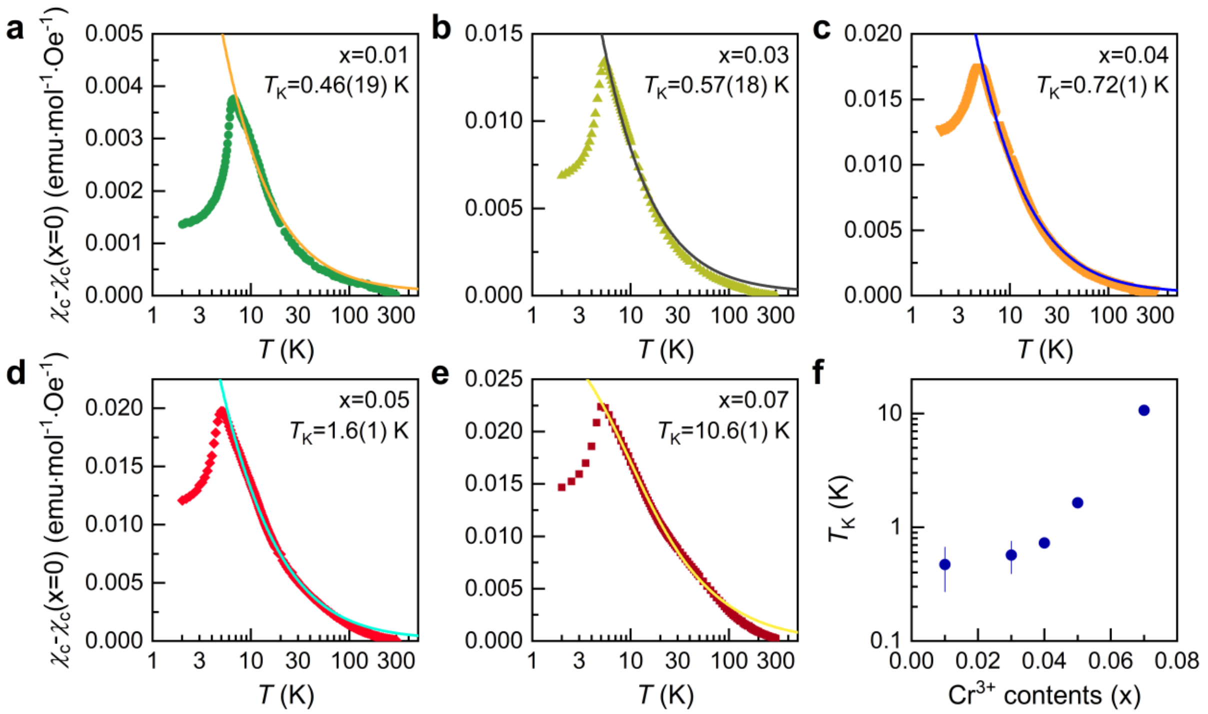

Supplementary Fig. 10 Comparison between the subtracted magnetic susceptibility and the theoretical impurity susceptibility of the equivalent multichannel Kondo effect. a-e Temperature dependence of the subtracted magnetic susceptibility for $B//c$=0.1 T in a semilog scale. The solid curves indicate the fits to the data using the theoretical impurity susceptibility of the equivalent multichannel Kondo effect. **f** Kondo temperature as a function of the Cr^{3+} contents. The error bars of T_K represent the uncertainty in a temperature window whether the logarithmic law is valid.

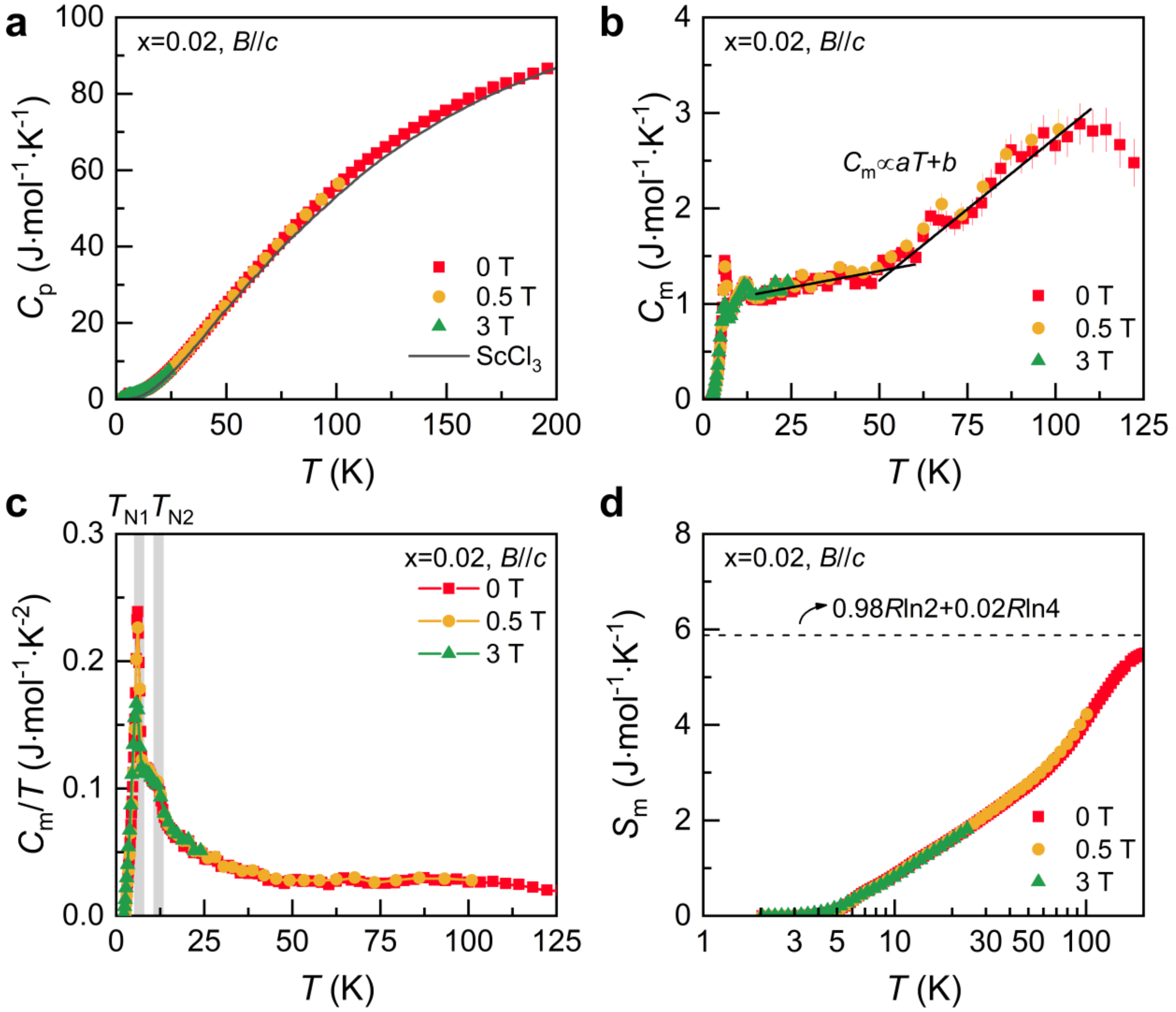

Supplementary Fig. 11 Thermodynamic properties of α-$Ru_{1-x}Cr_xCl_3$ (x=0.02). a Temperature dependence of the specific heat of α-$Ru_{1-x}Cr_xCl_3$ (x=0.02) at different fields and its lattice contribution. The lattice contribution to the specific heat is estimated from the isostructural nonmagnetic counterpart $ScCl_3$ by scaling with the molecular mass ratio $M_{\mathrm{Ru_{0.98}Cr_{0.02}Cl_3}}/M_{\mathrm{ScCl_3}}$ and the Debye temperature. **b** Magnetic specific heat as a function of temperature. The solid lines represent the linear dependence of $C_m(T)$. The error bars correspond to one standard deviation of the repeated specific data points. **c**, Magnetic specific heat divided by temperature vs. temperature. The grey vertical bars indicate the two magnetic transition temperatures T_{N1}=6 K and T_{N2}=12 K related to stacking faults. **d** Field and temperature dependence of the magnetic entropy calculated from $S_m(T) = \int C_m/T\, dT$.

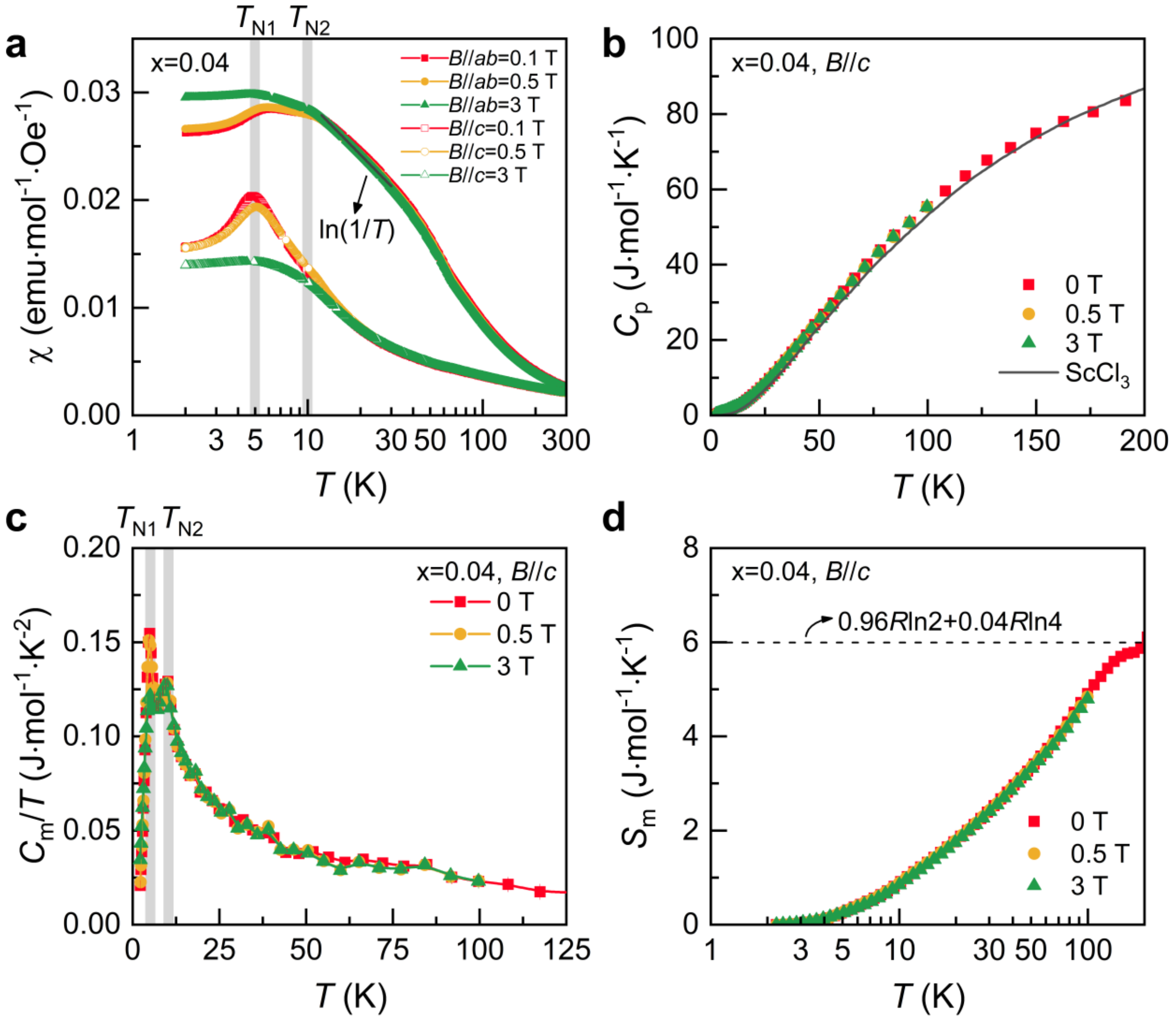

Supplementary Fig. 12 Magnetic susceptibility and specific heat of *α*-Ru$_{1\text{-}x}$Cr$_x$Cl$_3$ (*x*=0.04). **a** Temperature dependence of the static magnetic susceptibility under various applied fields along the *ab*-plane (closed symbols) and *c*-axis (open symbols) in a semi-log scale. The antiferromagnetic ordering at T_N=5.97 K for *B*//*ab* (4.86 K for *B*//*c*) is gradually suppressed with increasing field. The grey vertical bars indicate the two magnetic transition temperatures T_{N1}=4.8 K and T_{N2}=10.4 K related to stacking faults. The solid line represents a logarithmic dependence of χ_{ab}. **b** Temperature dependence of the specific heat of *α*-Ru$_{1\text{-}x}$Cr$_x$Cl$_3$ (*x*=0.04) at different fields and its lattice contribution. The lattice contribution to the specific heat is estimated from the isostructural nonmagnetic counterpart ScCl$_3$ by scaling the molecular mass ratio $M_{\mathrm{Ru_{0.96}Cr_{0.04}Cl_3}}/M_{\mathrm{ScCl_3}}$ and the Debye temperature. **c** Magnetic specific heat divided by temperature vs. temperature. The error bars correspond to one standard deviation of the repeated specific data points. **d** Field and temperature dependence of the magnetic entropy calculated from $S_m(T) = \int C_m/T\,dT$.

Supplementary Note 4. Weak transverse field μSR results of α-$Ru_{1-x}Cr_xCl_3$ (x=0.04)

We summarize the weak transfer field (wTF) μSR data in Supplementary Fig. 13. In the wTF-μSR experiments, the long-range ordering would give rise to a loss of initial asymmetry (t=0) or a rapidly relaxing component without oscillation signal due to the established static local field that prevails over the applied wTF. As expected, we observe the development of the fast-relaxing component and its enhancement with decreasing temperature. For quantitative analysis, the wTF spectra are fitted with a sum of an exponentially decaying cosine and a simple exponential function (Methods). The obtained parameters are plotted in Supplementary Fig. 13b-e. We identify two characteristic temperatures T_{N1}=5 K and T_{N2}=12 K that indicate the long-range magnetic order of ABC- and AB-type stacking patterns, consistent with the thermodynamic results in Supplementary Fig. 12.

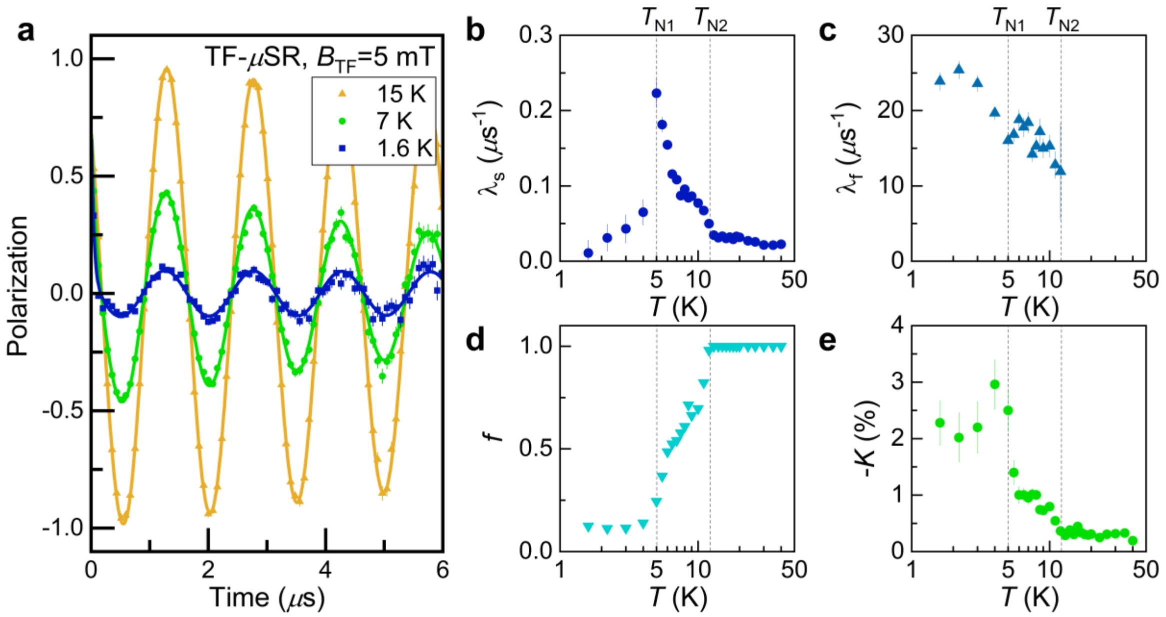

Supplementary Fig. 13 Weak transverse field μSR results of α-$Ru_{1-x}Cr_xCl_3$ (x=0.04). a Representative wTF-μSR spectra at selective temperatures. **b,c** Temperature dependence of the slow and fast muon spin relaxation rate in a semilog scale, λ_s and λ_f. $\lambda_s(T)$ displays a λ-like peak T_N=5 K, pointing out the antiferromagnetic ordering. **d** Slow relaxing fraction f as a function of temperature in a semilog scale. **e** Temperature dependence of the muon Knight shift K in a semilog scale. The vertical dashed lines indicate the characteristic temperatures T_N=5 K and T_{N2}=12 K. The error bars of the wTF spectra correspond to the square root of the total number of detected positrons resulting from muon decays. Error bars of the relaxation rate λ, the relaxing fraction, and the muon Knight shift K represent one standard deviation of the fit parameters.

Supplementary Note 5. μSR results of α-$Ru_{1-x}Cr_xCl_3$ (x=0.04)

In Supplementary Fig. 14 and Fig. 15, we present zero-field and high transverse-field μSR spectra of α-$Ru_{1-x}Cr_xCl_3$ (x=0.04). In order to determine the inhomogeneous magnetism composed of quasistatic and dynamic magnetism, we further carried out the longitudinal field (LF) μSR experiments at T=1.5 K. As displayed in Supplementary Fig. 16, LF-μSR spectra systematically shift upward with increasing field and are nearly fully polarized at B_{LF}=200 mT. This decoupling field suggests that the local static field is estimated to be $\langle B_{loc} \rangle \sim$20 mT, which is one-tenth of 200 mT. Nevertheless, we observe a small but discernible weak relaxation at H_{LF}=200 mT, corroborating the coexisting quasistatic and dynamic magnetism in the ground state. The LF-μSR spectra are fitted with a sum of the static and dynamic Gaussian Kubo-Toyabe functions (Methods). From the fittings, the local static field is evaluated to be $\langle B_{loc} \rangle \sim$16.88 mT, comparable to the roughly estimated value above.

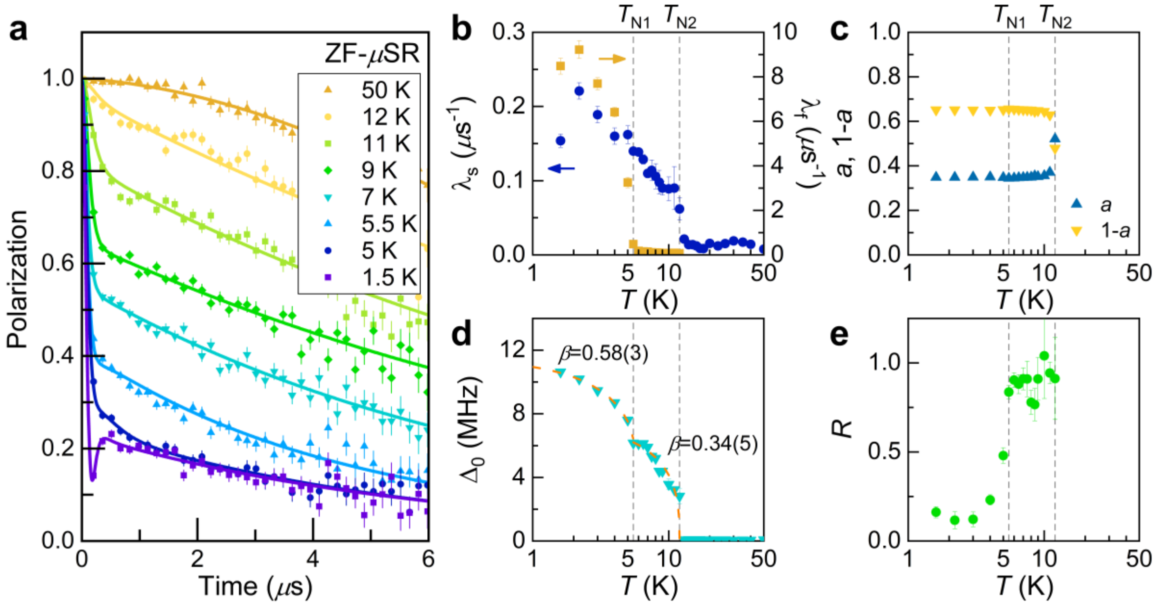

Supplementary Fig. 14 Zero-field μSR results of α-$Ru_{1-x}Cr_xCl_3$ (x=0.04). a Representative ZF-μSR spectra at selected temperatures. The solid lines are the fittings described in Methods. **b** Temperature dependence of the slow (λ_s) and fast (λ_f) muon spin relaxation rate on a semi-log scale. **c** Temperature dependence of the tail fraction (a) and the damped relaxing fraction (1-a) in the Gaussian-broadened-Gaussian function. **d** Mean value of the internal Gaussian field distribution Δ_0 as a function of temperature in a semi-log scale. The dashed curves are the fittings to the order parameter behaviors $\Delta_0(T)=\Delta_0(T=0\ \mathrm{K})[1-(T/T_{N1})]^{\beta}$ and $\Delta_0(T)=\Delta_0(T=0\ \mathrm{K})[1-(T/T_{N2})]^{\beta}$. **e** Temperature dependence of the relative Gaussian width $R=W/\Delta_0$. The vertical dashed lines indicate the characteristic temperatures T_{N1}=5 K and T_{N2}=12 K. The error bars of

the ZF-μSR spectra represent the square root of the total number of detected positrons resulting from muon decays. Error bars of the relaxation rate λ, Δ_0, and the relative Gaussian width R are one standard deviation of the fit parameters.

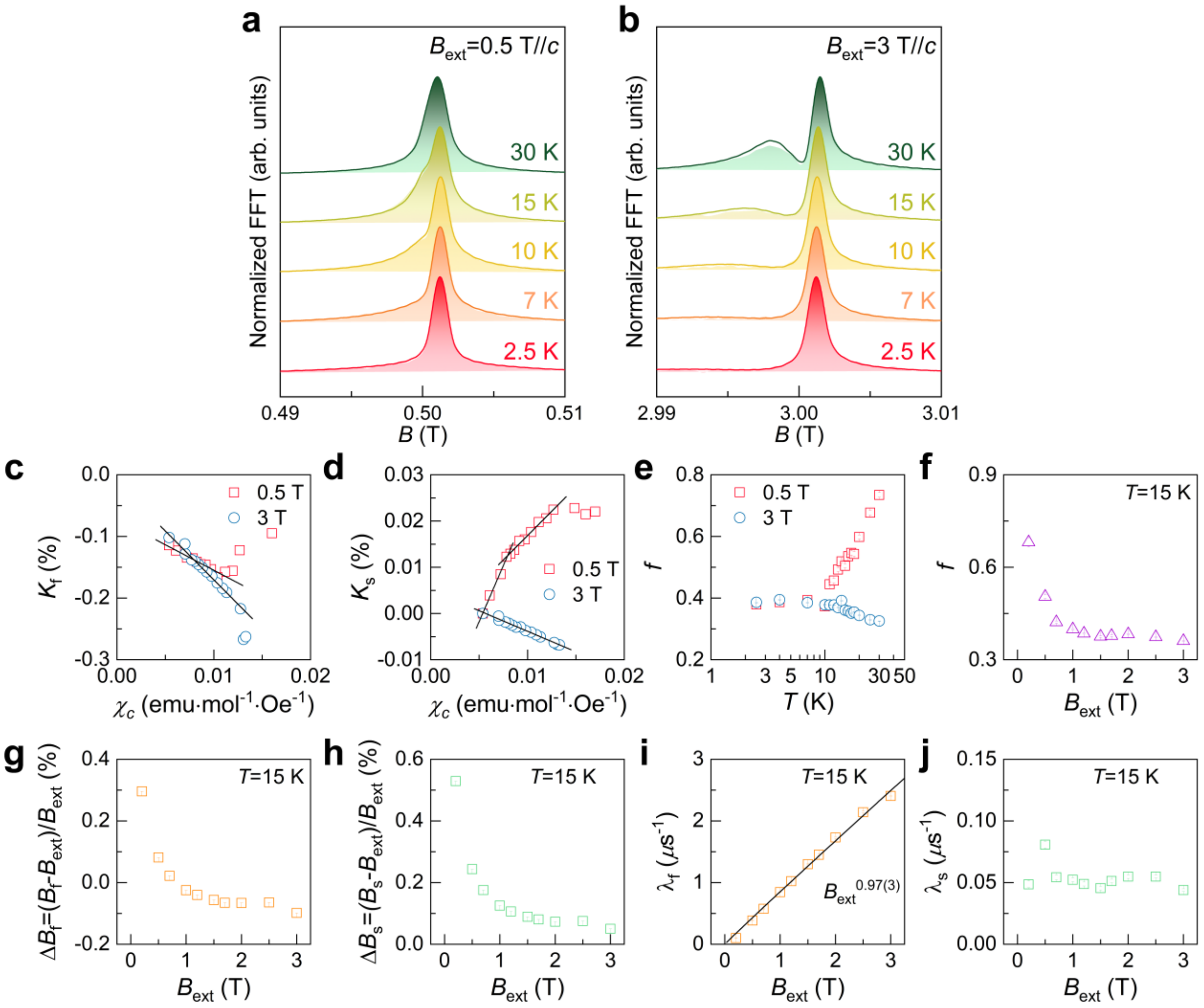

Supplementary Fig. 15 High transverse-field μSR spectra and field-dependent parameters of α-Ru$_{1-x}$Cr$_x$Cl$_3$ (x=0.04). a,b Normalized FFT amplitudes of hTF-μSR in applied fields of $B_{ext}//c$=0.5 and 3 T at different temperatures. The solid curves represent the fitting results with two Lorentzian decaying cosine functions (Methods). The data are vertically shifted for clarity. **c,d** Fast and slow muon Knight shift (K_f and K_s) vs. the dc magnetic susceptibility χ_c measured at identical magnetic fields. The solid lines denote the fittings using the relation $K_{f,s}=K_0+A_{hf}\chi_c$, where K_0 is the temperature-independent shift and A_{hf} is the hyperfine coupling constant. The hyperfine coupling constants are evaluated to be -0.082 (7) T/μ_B and -0.157(5) T/μ_B for B_{ext}=0.5 and 3 T in K_f, and 0.048(2) T/μ_B, 0.021(1) T/μ_B, -0.0088(2) T/μ_B for B_{ext}=0.5, 0.5, and 3 T in K_s, respectively. **e** Temperature dependence of the slow relaxing fraction. **f** Slow relaxing fraction as a function of field. **g,h** Field dependence of the field shift for the fast and slow component (ΔB_f and ΔB_s) in the Kondo regime (T=15 K). **i,j**

Fast and slow muon relaxation rate (λ_f and λ_s) versus B_{ext} in the Kondo regime. The solid curve denotes the power-law behavior $\lambda_f \sim B_{ext}^{0.97(3)}$. The vertical dashed line represents the crossover field. With increasing field, $\lambda_s(B_{ext})$ is nearly field independent, while $\lambda_f(B_{ext})$ monotonically increases. The error bars of the relaxation rate λ, the relaxing fraction, and the field shift correspond to one standard deviation from the χ^2 fit.

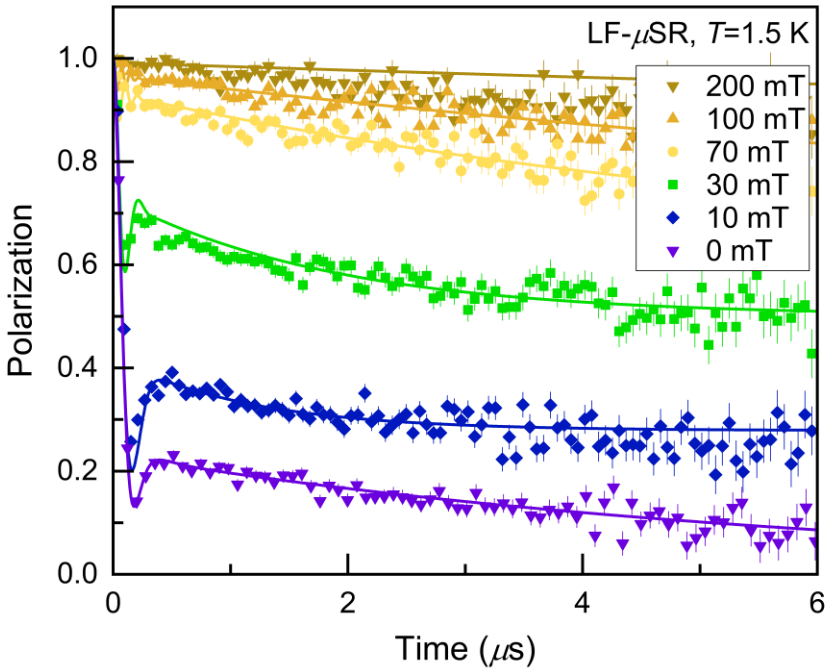

Supplementary Fig. 16 Longitudinal field μSR results of α-$Ru_{1-x}Cr_xCl_3$ (x=0.04). LF-μSR spectra at T=1.5 K. The solid curves represent the fits to the data using a sum of the static and dynamic Gaussian Kubo-Toyabe functions. The error bars of the LF- μSR spectra correspond to the square root of the total number of detected positrons.

Supplementary references

1. Roslova, M. *et al*. Detuning the honeycomb of the α-$RuCl_3$ Kitaev lattice: A Case of Cr^{3+} dopant. Inorg. Chem. **58**, 6659 (2019).

2. Glamazda, A., Lemmens, P., Do, S.-H., Kwon, Y. S. & Choi, K.-Y. Relation between Kitaev magnetism and structure in α−$RuCl_3$. Phys. Rev. B **95**, 174429 (2017).

3. Mai, T. T., McCreary, A., Lampen-Kelley, P., Butch, N., Simpson, J. R., Yan, J.-Q., Nagler, S.E., Mandrus, D., Hight Walker, A. R., & Valdes Aguilar, R. Polarization-resolved Raman spectroscopy of α−$RuCl_3$ and evidence of room-temperature two-dimensional magnetic scattering. Phys. Rev. B **100**, 134419 (2019).

4. Sears, J. A., Zhao, Y., Xu, Z., Lynn, J. W. & Kim, Y.-J. Phase diagram of $\alpha-RuCl_3$ in an in-plane magnetic field. Phys. Rev. B **95**, 180411 (2017).

5. Schlottmann, P. & Sacramento, P. D. Multichannel Kondo problem and some applications. Adv. Phys. **42**, 641 (1993).

6. Desgranges, H.-U. Thermodynamics of the n-channel Kondo problem (numerical solution). J. Phys. C: Solid State Phys. **18**, 5481 (1985).

7. Vojta, M., Mitchell, A. K. & Zschocke, F. Kondo Impurities in the Kitaev Spin Liquid: Numerical Renormalization Group Solution and Gauge-Flux-Driven Screening, Phys. Rev. Lett. **117**, 037202 (2016).
